# Supplementary material for: Unraveling 5f-6d hybridization in uranium compounds via spin-resolved L-edge spectroscopy
Source: Nat Commun. 2017 Oct 31;8:1203. doi: 10.1038/s41467-017-01524-1 (PMC5662594; doi:10.1038/s41467-017-01524-1)
Supplement: Supplementary file 1 — Supplementary Information [file 41467_2017_1524_MOESM1_ESM.pdf]

Supplementary Note 1: Sample preparation

Supplementary Table 1: **Structural parameters of UCu_2Si_2 and UMn_2Si_2.** Lattice parameters and crystallographic space group of the two systems are listed as obtained from Rietveld refinement of powder diffraction measurements at room temperature of the samples.

Compound	UCu_2Si_2	UMn_2Si_2
Structure	$ThCr_2Si_2$-type	$ThCr_2Si_2$-type
Space Group	I4/mmm	I4/mmm
a,b/*oA*	3.980	3.961
c/*oA*	9.937	9.512
V/*oA*^{3}	157.93	149.329

Polycrystalline samples of UMn_2Si_2 and UCu_2Si_2 were prepared by arc melting the high purity elements (U=99.9%, Mn, Cu and Si=99.9999%) in argon atmosphere followed by an annealing at 800°C for five days in an evacuated quartz ampoule. The X-ray diffraction pattern confirms the single-phase formation of the compounds. The evaluated lattice parameters and unit cell volume are shown in Supplementary Table 1, which were obtained using a Rietveld profile fit.

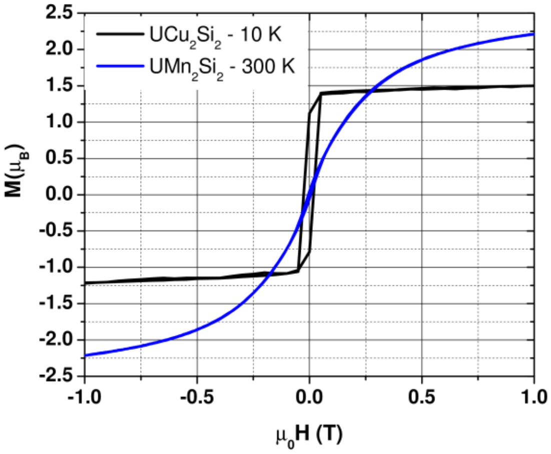

Supplementary Figure 1: **Magnetization curves of UCu_2Si_2 and UMn_2Si_2**. Field dependence of bulk magnetization for UCu_2Si_2 and UMn_2Si_2 at 10 and 300 K, respectively.

The bulk magnetic properties of the samples used in our XMCD experiments were measured in a Quantum Design MPMS magnetometer. The field dependence of the magnetization at the corresponding temperature of the XMCD experiment is shown in Supplementary Fig. 1.

Supplementary Note 2: Theory of X-ray absorption spectroscopy

The detailed description about the theory of the XAS process is given in the article of Galera et. al [1]. We used a LSDA+U approach in a mono-electronic description. In this approach, the normalized atomic absorption cross section for absorption or XMCD is given by the Fermi's golden rule:

$$\sigma = 4\pi^2\alpha\hbar\omega \sum_{fi} |< \psi_f|\mathbf{O}|\psi_i >|^2 \delta(\hbar\omega - (E_f - E_i)) \qquad (1)$$

where ψ_i and ψ_f are respectively the ground and photo-excited states, **O** the interacting operator between the electromagnetic field and the material, $\hbar\omega$ the photon energy and $\delta(\hbar\omega - (E_f - E_i))$ the state density.

In our systems there is only one atom of Uranium in the unit cell, thus no summation on atoms or symmetry operation must be added to the previous formula to get the unit mesh cross section. In the X-ray regime, the magnetic part of the electromagnetic field can be neglected and the operator **O** is reduced to its electric part. The electric part of the operator can be written through the multipolar expansion of this electric field up to the quadrupolar term as:

$$\mathbf{O} = \boldsymbol{\varepsilon}.\mathbf{r}\left(1 + \frac{1}{2}\mathrm{i}\mathbf{k}.\mathbf{r}\right) \qquad (2)$$

where $\mathbf{r}$ is the electron position measured from the absorbing ion, $\boldsymbol{\varepsilon}$ the polarization of the photon and $\mathbf{k}$ its corresponding wavevector. The quadrupolar term of the operator is negligible below 5 keV, and increase with the energy (E). since $|\mathbf{k}| = \frac{2\pi}{\lambda} = \frac{2\pi E}{hc}$, where λ is the wavelength, h is the Plank constant and c the speed of light. Thanks to the selection rules, at the L_3 edge, the quadrupolar term probes the *f* and *p* states whereas the dipole probes the *d* and s states.

Supplementary Note 3: Density of states

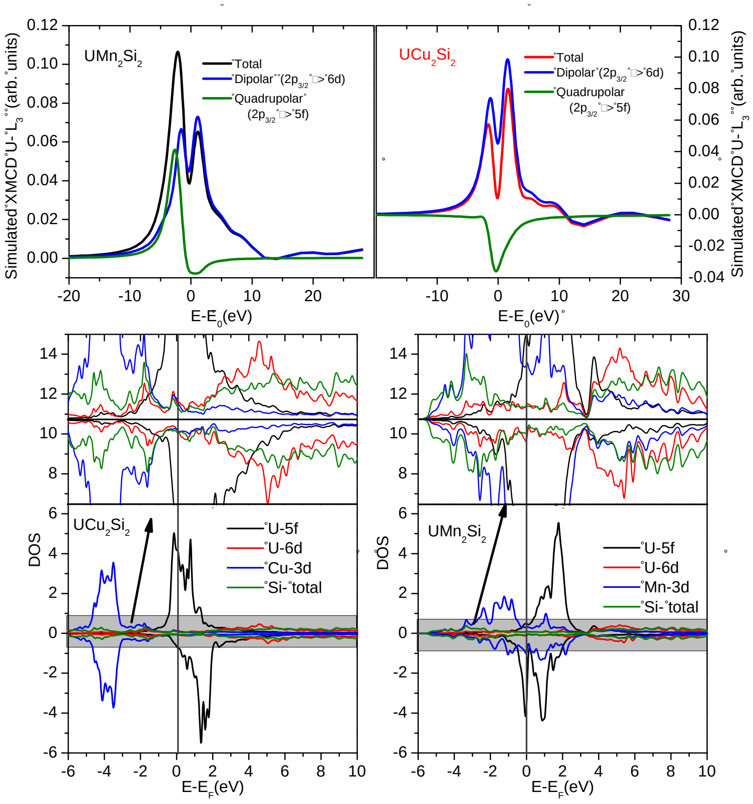

Supplementary Figure 2: **UMn_2Si_2 and UCu_2Si_2 theoretical simulations**. Dipolar and quadrupolar contributions to the L_3-edge XMCD determined using the FDMNES code, as well as the density of states determined by LDA+U calculations.

Density functional theory ab initio calculations were performed using the WIEN2K implementation of the full-potential linearized augmented plane-wave (APW) method with a double-counting scheme and the rotationally invariant local density approximation LDA+U functional with U = 1.25 eV and including spin-orbit coupling [2] for the easy axis magnetization directions as reported in the literature [3,4]. More detailed parameters for these simulations have already being reported in the literature [5].

LDA+U calculations of the energy and spin dependent density of states corresponding to the UMn_2Si_2 and UCu_2Si_2 are shown in Supplementary Fig. 2. While the 6*d* orbitals are almost equivalent in both compounds, the 5*f* orbitals present an inversion between the spin up and down occupancy. This result is consistent with the argument that while the 5*f* and 6*d* orbitals are parallel for the UMn_2Si_2 compound, they are anti-parallel for the UCu_2Si_2, as discussed in the main text in view of the experimental XMCD data. These simulations also shed some light regarding

the contribution from interactions with Silicon orbitals. The hybridization of Si orbitals with those of others elements of the material is almost the same for both compounds. However, the induced magnetic moment in the Si atoms is about one hundred times lower than the magnetic moment observed for the Uranium. This make us to believe that, for at least these compounds, the magnetic contribution from the Si atom can be completely neglected in the interpretation of the changes of the L_3-edge XMCD signal.

Supplementary Note 4: XMCD at K edge of Mn and Cu

In order to demonstrate the importance of the hybridization bettween U-6d/Mn/Cu-3d we had measured the Mn and Cu K-edge XMCD (as shown in Supplementary Fig. 3) to give preliminary information about the orbital magnetism of the 3d lattice.). We note that this observation is not a proof that Cu ions are magnetic, but indicate that one considerable amount of orbital moment is transferred to the 4p-3d Cu orbitals.

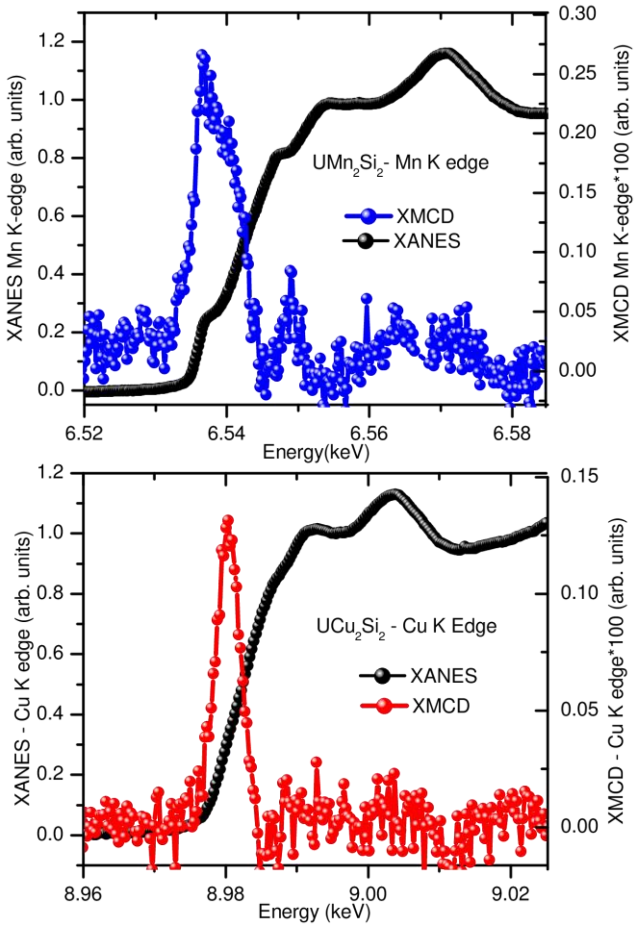

Supplementary Figure 3: **Mn/Cu K-edge spin-dependent X-ray absorption spectroscopy**. Normalized XANES signal across the Mn and Cu K edges on UT_2Si_2 samples are presented in black curves. The XANES spectra were normalized to 1 at energies well above the edges. Corresponding normalized XMCD data for μ_0H = 1 T measured at 10 K for Cu edge and at 300 K to Mn edge are presented in red (UCu_2Si_2) and blue (UMn_2Si_2).

Supplementary Note 5: Table data for temperature dependence uranium L_3-edge XMCD

On Supplementary Tables 2 and 3 we present the complete experimental data for L_3-edge XMCD of the the UMn_2Si_2 compound at 300 K and 22 K, respectively.

Supplementary Table 2: **Experimental L_3-edge data for UMn_2Si_2 at 300 K**. Normalized XMCD data as a function of energy as shown in Figure 2 (main text) for the UMn_2Si_2 at 300 K.

UMn2Si2 300													
Energy (eV)	XMCD*100	Energy (eV)	XMCD*100	Energy (eV)	XMCD*100	Energy (eV)	XMCD*100	Energy (eV)	XMCD*100	Energy (eV)	XMCD*100	Energy (eV)	XMCD*100
16995.08	7.53E-04	17062.26	5.94E-04	17131.16	0.00792	17200.06	-0.00946	17268.96	-0.00327	17337.86	0.00212	17389.53	0.00608
16995.94	0.00299	17063.12	-0.00435	17132.02	-0.00382	17200.92	-0.01244	17269.82	-0.00521	17338.72	-0.00873	17390.39	0.00595
16996.80	-0.00589	17063.98	-0.00733	17132.88	-0.00812	17201.78	-0.01753	17270.68	0.00418	17339.58	-0.00913	17391.25	3.50E-04
16997.66	0.00183	17064.84	-0.00693	17133.74	-0.00602	17202.64	-0.01445	17271.54	-0.00767	17340.44	-0.01304	17392.12	-0.0025
16998.52	-0.00478	17065.70	-0.00414	17134.60	-0.01121	17203.50	-0.00679	17272.40	-0.00898	17341.30	-0.0211	17392.98	-0.0079
16999.39	0.00472	17066.56	-0.00437	17135.46	-0.00453	17204.36	-0.00495	17273.26	-0.01182	17342.16	-0.00875	17393.84	-0.01048
17000.25	0.00731	17067.42	-0.0091	17136.32	-0.00488	17205.22	-0.01372	17274.12	-0.00914	17343.02	-0.01165	17394.70	-0.01758
17001.11	-0.00364	17068.29	-0.01763	17137.19	0.00671	17206.09	-0.0231	17274.99	-0.01075	17343.89	-0.01121	17395.56	-0.00221
17001.97	-0.00113	17069.15	-0.01618	17138.05	-0.00638	17206.95	-0.00344	17275.85	-0.00725	17344.75	5.79E-04	17396.42	0.0173
17002.83	-0.01628	17070.01	-0.01269	17138.91	-0.00476	17207.81	-0.00577	17276.71	-0.02033	17345.61	-1.51E-05	17397.28	-0.0102
17003.69	0.01279	17070.87	-0.00602	17139.77	-0.00353	17208.67	-0.01187	17277.57	-0.02629	17346.47	0.00463	17398.14	-0.0032
17004.55	0.01463	17071.73	-0.00693	17140.63	-0.00406	17209.53	-0.0224	17278.43	-0.00941	17347.33	0.0098	17399.01	0.00682
17005.41	0.01526	17072.59	-0.00796	17141.49	0.00144	17210.39	-0.00147	17279.29	-0.00104	17348.19	-0.00262	17399.87	0.01799
17006.28	0.00862	17073.45	-0.00721	17142.35	0.00747	17211.25	-0.00136	17280.15	-0.01488	17349.05	-0.01008	17400.73	0.01748
17007.14	-0.00156	17074.31	-0.0104	17143.21	5.67E-04	17212.11	-0.01821	17281.01	-0.01442	17349.91	-0.00526	17401.59	0.00775
17008.00	0.00103	17075.18	-0.0111	17144.08	0.01123	17212.98	-0.01431	17281.88	-0.01923	17350.78	0.01507	17402.45	0.00226
17008.86	-0.00602	17076.04	-0.00844	17144.94	0.01714	17213.84	-0.01348	17282.74	-0.01469	17351.64	0.00887	17403.31	0.01309
17009.72	0.00603	17076.90	0.00375	17145.80	0.01976	17214.70	-0.01333	17283.60	-0.0101	17352.50	-0.00305	17404.17	-0.00658
17010.58	-5.62E-04	17077.76	-0.00841	17146.66	0.02682	17215.56	-0.01721	17284.46	-0.01855	17353.36	-0.0082	17405.03	-0.0113
17011.44	-0.01669	17078.62	-0.00364	17147.52	0.01242	17216.42	-0.0226	17285.32	-0.01178	17354.22	-0.01629	17405.90	0.02029
17012.30	0.0039	17079.48	-0.0178	17148.38	0.01772	17217.28	-0.00772	17286.18	-0.01886	17355.08	0.00596	17406.76	0.00865
17013.17	-0.00102	17080.34	-0.01019	17149.24	0.0178	17218.14	-0.01214	17287.04	-0.0283	17355.94	0.00491	17407.62	0.0137
17014.03	-0.00945	17081.20	-0.00669	17150.10	0.02347	17219.00	-0.00479	17287.90	-0.02403	17356.80	0.00507	17408.48	0.02956
17014.89	-0.00507	17082.07	-0.01015	17150.97	0.02719	17219.87	-0.02665	17288.77	-0.00139	17357.67	8.20E-04	17409.34	0.01905
17015.75	0.00674	17082.93	-0.01352	17151.83	0.03851	17220.73	-0.01461	17289.63	-0.02177	17358.53	0.00367	17410.20	-0.01367
17016.61	-0.01095	17083.79	-0.00966	17152.69	0.05193	17221.59	0.01275	17290.49	-0.02037	17359.39	-0.00481	17411.06	-0.00116
17017.47	0.01385	17084.65	-0.01596	17153.55	0.06862	17222.45	0.01046	17291.35	-0.0215	17360.25	-4.97E-04	17411.92	-0.00884
17018.33	0.01272	17085.51	3.83E-04	17154.41	0.06731	17223.31	-0.00315	17292.21	-0.00835	17361.11	0.00639	17412.79	0.0069
17019.19	0.00899	17086.37	-0.00913	17155.27	0.09474	17224.17	-0.00225	17293.07	7.86E-04	17361.97	-0.01346	17413.65	0.00465
17020.06	-0.00333	17087.23	-0.00452	17156.13	0.1113	17225.03	0.00579	17293.93	0.00446	17362.83	-0.00656	17414.51	0.00295
17020.92	0.00653	17088.09	-0.00527	17156.99	0.12876	17225.89	0.00156	17294.79	-0.00487	17363.69	-0.01167	17415.37	-0.01304
17021.78	-0.00775	17088.96	-0.01743	17157.86	0.12678	17226.76	-0.00468	17295.66	-0.00159	17364.56	-0.01148	17416.23	-0.01029
17022.64	0.00444	17089.82	-0.01532	17158.72	0.1305	17227.62	0.00108	17296.52	-0.0154	17365.42	0.00214	17417.09	-0.00382
17023.50	0.01253	17090.68	-0.00461	17159.58	0.12426	17228.48	-0.00364	17297.38	-0.01337	17366.28	0.00921	17417.95	-0.01105
17024.36	-0.00514	17091.54	-0.0034	17160.44	0.11716	17229.34	-0.00497	17298.24	-0.01542	17367.14	0.02351	17418.81	-0.00715
17025.22	-0.00535	17092.40	-0.00666	17161.30	0.09803	17230.20	-0.00269	17299.10	-0.0206	17368.00	-0.00456	17419.68	-0.00238
17026.08	-1.40E-04	17093.26	-0.00226	17162.16	0.09871	17231.06	0.00217	17299.96	-0.01188	17368.86	-0.01459	17420.54	0.01439
17026.95	-6.37E-05	17094.12	-1.76E-04	17163.02	0.10258	17231.92	8.49E-04	17300.82	-0.0135	17369.72	2.19E-05	17421.40	0.00284
17027.81	0.00874	17094.98	6.40E-04	17163.88	0.10224	17232.78	0.0044	17301.68	-0.01427	17370.58	0.01105	17422.26	0.01233
17028.67	0.0061	17095.85	-0.00155	17164.75	0.09773	17233.65	0.00383	17302.55	-0.01082	17371.45	0.01108	17423.12	0.01205
17029.53	-0.0059	17096.71	-0.02079	17165.61	0.08722	17234.51	-0.00369	17303.41	-0.01179	17372.31	-0.00354	17423.98	-0.01275
17030.39	0.00677	17097.57	-0.01298	17166.47	0.07786	17235.37	-0.0152	17304.27	-0.02583	17373.17	0.00797	17424.84	-0.0059
17031.25	-0.00601	17098.43	-0.01348	17167.33	0.06733	17236.23	-0.00581	17305.13	-0.02181	17374.03	0.00833	17425.70	0.01493
17032.11	-0.00505	17099.29	-0.00961	17168.19	0.05733	17237.09	0.00632	17305.99	-0.02916	17374.89	0.01433	17426.57	0.00147
17032.97	0.0013	17100.15	-0.01211	17169.05	0.04387	17237.95	0.009	17306.85	-0.03491	17375.75	0.01893	17427.43	0.01466
17033.84	-0.01199	17101.01	-0.00613	17169.91	0.04216	17238.81	0.00164	17307.71	-0.01721	17376.61	0.01263	17428.29	0.0042
17034.70	-3.46E-04	17101.87	0.0019	17170.77	0.0347	17239.67	-0.00171	17308.57	-0.00471	17377.47	0.00781	17429.15	0.00778
17035.56	-2.98E-04	17102.74	-0.00176	17171.64	0.0291	17240.54	7.30E-04	17309.44	-0.00318	17378.34	0.00568	17430.01	0.01535
17036.42	-0.01054	17103.60	-0.01493	17172.50	0.02741	17241.40	-0.01457	17310.30	-0.00845	17379.20	0.01055	17430.87	-6.20E-04
17037.28	-0.01912	17104.46	-0.02889	17173.36	0.02291	17242.26	-0.00903	17311.16	-0.00753	17380.06	0.01558	17431.73	-0.01279
17038.14	-0.00806	17105.32	-8.28E-04	17174.22	0.00559	17243.12	-0.00457	17312.02	-0.01268	17380.92	0.00578	17432.59	-0.0093
17039.00	-0.01479	17106.18	0.00849	17175.08	0.01325	17243.98	-0.00314	17312.88	-0.01157	17381.78	0.01156	17433.46	1.44E-04
17039.86	0.00254	17107.04	-0.00411	17175.94	-0.00208	17244.84	0.00391	17313.74	-0.00136	17382.64	-0.03498	17434.32	0.00347
17040.73	0.00675	17107.90	-0.00945	17176.80	-0.00328	17245.70	-0.00654	17314.60	-0.01306	17383.50	-0.01934	17435.18	0.00348
17041.59	0.00371	17108.76	-0.00501	17177.66	-0.0161	17246.56	-0.00334	17315.46	-0.00448	17384.36	0.00257	17436.04	-0.00646
17042.45	0.00506	17109.63	-0.00795	17178.53	-0.01219	17247.43	-0.03321	17316.33	-0.00482	17385.23	0.01117	17436.90	-0.0076
17043.31	0.0029	17110.49	-0.0108	17179.39	0.00198	17248.29	-0.03129	17317.19	-0.00382	17386.09	0.00151	17437.76	0.00544
17044.17	-0.01047	17111.35	-0.0023	17180.25	0.00789	17249.15	-0.01949	17318.05	-0.00576	17386.95	0.00393		
17045.03	-0.00521	17112.21	-0.00696	17181.11	-0.0086	17250.01	-0.01075	17318.91	-0.01139	17387.81	0.00429		
17045.89	-0.00741	17113.07	-0.00781	17181.97	-0.00657	17250.87	-0.00959	17319.77	-0.00512	17388.67	0.00204		
17046.75	-0.00497	17113.93	-0.02432	17182.83	-0.01445	17251.73	0.00308	17320.63	-0.00273				
17047.62	0.00461	17114.79	-0.02148	17183.69	-0.01296	17252.59	1.89E-04	17321.49	0.00436				
17048.48	-0.00699	17115.65	6.46E-04	17184.55	-0.00167	17253.45	0.00161	17322.35	-0.00486				
17049.34	-0.00925	17116.52	0.00971	17185.42	-0.00152	17254.32	0.0195	17323.22	-0.01075				
17050.20	-0.01074	17117.38	8.94E-04	17186.28	0.00448	17255.18	0.0072	17324.08	-0.00973				
17051.06	0.00196	17118.24	0.00124	17187.14	0.01691	17256.04	0.00426	17324.94	-0.01769				
17051.92	-0.01181	17119.10	-0.00501	17188.00	0.00757	17256.90	0.00325	17325.80	-0.0256				
17052.78	-0.01636	17119.96	6.06E-04	17188.86	0.01005	17257.76	-0.00292	17326.66	-0.02656				
17053.64	-0.01702	17120.82	-3.24E-04	17189.72	0.02306	17258.62	0.00204	17327.52	-0.00713				
17054.51	-0.00347	17121.68	-0.0064	17190.58	0.01622	17259.48	-0.00104	17328.38	-0.00338				
17055.37	-0.00322	17122.54	-0.00158	17191.44	0.02343	17260.34	0.00477	17329.24	-0.00298				
17056.23	-0.00202	17123.41	0.00688	17192.31	0.0159	17261.21	-0.01036	17330.11	-0.00887				
17057.09	-0.00944	17124.27	-0.0092	17193.17	0.00472	17262.07	0.0013	17330.97	-0.02042				
17057.95	4.72E-04	17125.13	0.00154	17194.03	0.01131	17262.93	0.02292	17331.83	-0.01735				
17058.81	-0.00774	17125.99	5.63E-04	17194.89	0.00883	17263.79	0.01613	17332.69	-0.02075				
17059.67	-0.00878	17126.85	2.82E-04	17195.75	-0.00382	17264.65	0.00846	17333.55	-0.00223				
17060.53	-0.01511	17127.71	0.00868	17196.61	0.01691	17265.51	0.00743	17334.41	-0.01513				
17061.40	-0.00283	17128.57	0.02126	17197.47	-0.00934	17266.37	-0.01346	17335.27	-0.01083				
		17129.43	0.00943	17198.33	0.0096	17267.23	-0.01244	17336.13	-0.01871				
		17130.30	0.00526	17199.20	0.00665	17268.10	-0.00334	17337.00	-0.01517				

Supplementary Table 3: **Experimental L_3-edge data for UMn_2Si_2 at 22 K**. Normalized XMCD data as a function of energy as shown in Figure 2 (main text) for the UMn_2Si_2 at 22 K.

UMn2Si2 22 K

Energy (eV)	XMCD*100	Energy (eV)	XMCD*100	Energy (eV)	XMCD*100	Energy (eV)	XMCD*100	Energy (eV)	XMCD*100	Energy (eV)	XMCD*100
17007.34	-0.0146058	17055.55	-0.023322	17105.07	-0.0141434	17154.59	0.198	17204.12	-0.0104853	17253.65	0.045463
17008.00	0.00807029	17056.20	-0.0145055	17105.73	-0.014489	17155.26	0.233	17204.78	-0.023291	17254.30	0.035915
17008.66	-0.00787907	17056.87	-0.00613103	17106.39	-0.00115067	17155.91	0.203	17205.44	-0.0344572	17254.97	0.052256
17009.32	-0.0245403	17057.52	-0.0163554	17107.05	-0.0217617	17156.58	0.243	17206.10	-0.0388867	17255.63	0.058171
17009.98	-0.00811545	17058.19	-0.0241006	17107.71	-0.016327	17157.24	0.26	17206.76	-0.0115626	17256.29	0.0335156
17010.64	0.0127188	17058.84	0.00310159	17108.37	-0.0142258	17157.90	0.261	17207.42	0.000306417	17256.95	0.0467315
17011.30	-0.00888059	17059.51	0.0156277	17109.03	0.0105986	17158.56	0.249	17208.08	-0.0247388	17257.61	0.0561156
17011.96	-0.00562444	17060.17	0.0283938	17109.69	-0.00660497	17159.22	0.25	17208.74	-0.035079	17258.27	0.0150688
17012.62	0.00740095	17060.83	0.000845118	17110.35	0.0155439	17159.88	0.243	17209.40	0.00583016	17258.93	0.0114685
17013.28	0.00180513	17061.49	0.0146651	17111.01	0.00781376	17160.54	0.242	17210.06	-0.00582289	17259.59	0.0302856
17013.94	0.00876062	17062.15	0.00578236	17111.67	0.0260014	17161.20	0.228	17210.72	-0.023704	17260.25	0.00906376
17014.60	0.00779967	17062.81	-0.000600202	17112.33	0.0158387	17161.86	0.227	17211.38	-0.0332627	17260.91	-0.0016361
17015.27	0.00085701	17063.47	0.00834805	17112.99	-0.00374768	17162.52	0.2	17212.05	-0.0398315	17261.57	-0.0185104
17015.92	-0.0170113	17064.13	0.0134447	17113.66	0.0128453	17163.18	0.188	17212.70	-0.00518586	17262.23	0.00567074
17016.59	-0.00459277	17064.79	0.00199935	17114.31	0.00802101	17163.84	0.172	17213.37	0.0414809	17262.89	0.00310652
17017.24	0.00691285	17065.45	0.00568031	17114.98	0.000193129	17164.50	0.17	17214.02	0.0103085	17263.55	0.00453027
17017.91	0.0132191	17066.11	-0.00285035	17115.63	0.00514091	17165.16	0.179	17214.69	0.0221691	17264.21	0.00191311
17018.56	0.00312711	17066.77	-0.0172836	17116.30	-0.0119793	17165.82	0.173	17215.34	0.0196069	17264.87	-0.0056709
17019.23	0.0176826	17067.43	0.00373791	17116.95	2.07608E-05	17166.48	0.144	17216.01	0.0207936	17265.53	-0.0014686
17019.88	-0.0181877	17068.09	0.0218289	17117.62	0.00693916	17167.14	0.132	17216.67	0.03358	17266.19	-0.0065016
17020.55	0.00974429	17068.75	-0.00954213	17118.27	0.0103741	17167.80	0.118	17217.33	0.040215	17266.85	-0.0092005
17021.20	0.0108703	17069.41	0.0215213	17118.94	-0.0152365	17168.46	0.0933997	17217.99	0.012462	17267.52	0.00216731
17021.87	0.00598813	17070.07	0.00475163	17119.59	0.0059537	17169.12	0.0742639	17218.65	0.00268013	17268.17	0.0209811
17022.52	-0.00697755	17070.73	0.0155197	17120.26	0.0172339	17169.78	0.0684748	17219.31	-0.00710704	17268.84	0.0146422
17023.19	-0.0351222	17071.39	-0.0219142	17120.91	0.00685292	17170.44	0.059831	17219.97	0.000786957	17269.49	0.0012871
17023.84	0.0279023	17072.05	-0.014509	17121.58	-0.00773105	17171.10	0.054126	17220.63	-0.00835097	17270.16	-0.0187895
17024.51	0.0260349	17072.71	-0.00895661	17122.24	0.0185411	17171.76	0.0456203	17221.29	-0.00212301	17270.82	-0.0074797
17025.17	0.00749721	17073.37	-0.0202064	17122.90	0.0126274	17172.42	0.036007	17221.95	0.0273221	17271.48	-0.0325309
17025.83	-0.0124326	17074.03	-0.0166589	17123.56	-0.000114048	17173.09	0.0082857	17222.61	0.0229046	17272.14	-0.006641
17026.49	-0.0128134	17074.69	-0.0175446	17124.22	-0.00486092	17173.74	0.00237042	17223.27	0.0414763	17272.80	-0.0038344
17027.15	0.0153406	17075.35	-0.0142119	17124.88	-0.0107628	17174.41	0.0310221	17223.93	-0.00263638	17273.46	-0.015707
17027.81	0.00331461	17076.01	-0.0118216	17125.54	-0.00369382	17175.06	0.0285354	17224.59	0.00103162	17274.12	-0.0017756
17028.47	-0.0103672	17076.67	0.00402587	17126.20	0.012048	17175.73	0.0219773	17225.25	0.0076827	17274.78	0.0148466
17029.13	-0.00867447	17077.33	0.0128586	17126.86	0.0143627	17176.39	0.00374311	17225.91	0.00803169	17275.44	0.0138039
17029.79	0.0182408	17077.99	0.00652569	17127.52	-0.00739187	17177.05	0.00739916	17226.57	0.000223268	17276.10	-0.0032413
17030.45	0.00164929	17078.66	0.000457339	17128.18	-0.0228743	17177.71	0.0280573	17227.23	0.0312589	17276.76	-0.0143407
17031.11	-0.0115731	17079.31	0.0170935	17128.84	-0.00966647	17178.37	0.0206031	17227.89	0.0264477	17277.42	0.00880503
17031.77	-0.00390146	17079.98	0.0170102	17129.50	-0.0072712	17179.03	0.00779165	17228.55	0.0101257	17278.08	-0.0284844
17032.43	0.00284677	17080.63	-0.0132365	17130.16	0.0033715	17179.69	0.0200479	17229.21	-0.000149149	17278.74	0.00238041
17033.09	0.0072414	17081.30	0.0174814	17130.82	0.0340782	17180.35	0.0209412	17229.87	0.0108156	17279.40	-0.0061965
17033.75	-0.00867531	17081.96	0.0213866	17131.48	0.00962612	17181.01	0.00157311	17230.54	-0.00706949	17280.06	0.00384982
17034.41	0.00869714	17082.62	0.0113681	17132.14	-0.0044779	17181.67	0.0325479	17231.19	0.0258325	17280.72	0.00457319
17035.07	0.000674915	17083.28	-0.0133877	17132.80	-0.00191088	17182.33	0.0156272	17231.86	0.0142462	17281.38	-0.0138267
17035.73	0.0163333	17083.94	-0.0178841	17133.46	0.00788807	17182.99	0.049233	17232.51	-0.0084004	17282.04	0.00459102
17036.39	0.0101833	17084.60	0.00883205	17134.13	0.00705733	17183.65	0.0333343	17233.18	0.000190292	17282.70	-0.0003078
17037.05	-0.00160523	17085.26	0.00168888	17134.78	0.00918166	17184.31	0.0313836	17233.84	0.0247818	17283.36	-0.0236295
17037.71	0.00466418	17085.92	0.00372635	17135.45	0.00397735	17184.97	0.024567	17234.50	0.0311524	17284.02	-0.0081946
17038.38	-0.0251895	17086.58	0.00449337	17136.11	0.0114917	17185.63	0.0171254	17235.16	0.0254162	17284.69	0.00535511
17039.03	-0.0223144	17087.24	-0.00821201	17136.77	0.00527474	17186.29	0.0349337	17235.82	-0.00696694	17285.34	-0.019027
17039.70	-0.00421489	17087.90	0.0159933	17137.43	0.0069594	17186.95	0.0568081	17236.48	-0.0179673	17286.01	-0.0126711
17040.35	-0.00933078	17088.56	-0.00545742	17138.09	0.00311136	17187.61	0.0324144	17237.14	0.00910092	17286.66	-0.002255
17041.02	-0.00133917	17089.22	-0.00372775	17138.75	-0.00105843	17188.27	0.0435056	17237.80	-0.00545272	17287.33	-0.0071246
17041.68	-0.000304768	17089.88	0.00503141	17139.41	-0.00150767	17188.93	0.0463879	17238.46	-0.0011911	17287.98	0.00931938
17042.34	-0.0139811	17090.54	-0.00346303	17140.07	-0.00524604	17189.59	0.0444691	17239.12	0.000849618	17288.65	-0.0050611
17043.00	-7.69117E-05	17091.20	-0.00403433	17140.73	0.00824814	17190.26	0.0277694	17239.78	-0.00851446	17289.31	-0.0021916
17043.66	-0.00415224	17091.86	0.00212599	17141.39	0.0100427	17190.91	0.0486385	17240.44	-0.0235322	17289.97	-0.04442
17044.32	-0.00408571	17092.52	0.0134034	17142.05	0.0454474	17191.58	0.0415231	17241.10	-0.0286588	17290.63	-0.0130264
17044.98	0.0236091	17093.18	0.00442759	17142.71	0.0323115	17192.23	0.0457348	17241.76	-0.0095934	17291.29	0.00171777
17045.64	-0.00854095	17093.84	0.000984864	17143.37	0.018685	17192.90	0.0428524	17242.42	-0.00939506		
17046.30	0.00918985	17094.50	-0.000970203	17144.03	0.0165179	17193.55	0.051534	17243.08	-0.0368701		
17046.96	0.0049919	17095.16	0.0148976	17144.69	0.0163264	17194.22	0.0471099	17243.74	0.00297474		
17047.62	-0.00116472	17095.83	0.00765794	17145.35	0.0300152	17194.88	0.0157079	17244.40	0.0168349		
17048.28	0.00751741	17096.48	0.0291986	17146.01	0.0229135	17195.54	0.0346466	17245.06	0.0348282		
17048.94	0.00909443	17097.15	-0.00611361	17146.67	0.0451464	17196.20	0.021009	17245.73	0.0462901		
17049.60	0.000357186	17097.80	0.0278109	17147.33	0.0454885	17196.86	0.0325228	17246.38	0.00722878		
17050.26	-0.016215	17098.47	0.0111367	17147.99	0.0453325	17197.52	0.00459941	17247.05	-0.00337368		
17050.92	0.00116843	17099.12	0.00823882	17148.65	0.0331122	17198.18	-0.0206446	17247.70	0.00360629		
17051.58	-0.00773957	17099.79	-0.0170466	17149.31	0.0509322	17198.84	0.00536693	17248.37	0.0448435		
17052.24	0.0207453	17100.45	-0.00606069	17149.98	0.0571963	17199.50	-0.0070178	17249.02	0.00965716		
17052.90	0.0202723	17101.11	0.00609619	17150.63	0.086255	17200.16	-0.0311748	17249.69	0.0147903		
17053.56	0.00553946	17101.77	0.0173135	17151.30	0.0935186	17200.82	-0.025201	17250.34	0.0304155		
17054.22	0.00367998	17102.43	0.0186434	17151.95	0.103	17201.48	-0.0058521	17251.01	0.0298514		
17054.88	0.00158511	17103.09	-0.0213754	17152.62	0.128	17202.14	-0.0339838	17251.66	0.0300195		
		17103.75	-0.00571327	17153.27	0.176	17202.80	-0.0358976	17252.33	0.0457343		
		17104.41	-0.00947142	17153.94	0.156	17203.46	-0.0315667	17252.98	0.0339009		

Supplementary References

[1] Galera, R.-M., Joly, Y., Rogalev, A. & Binggeli, N. The quadrupolar contribution to X-ray magnetic circular dichroism in REZn compounds. *Journal of Physics: Condensed Matter* **20**, 395217 (2008).

[2] Blaha, P., Schwarz, K., Madsen, G. K. H., Kvasnicka, D. & Luitz, J. *WIEN2k, An Augmented Plane Wave + Local Orbitals Program for Calculating Crystal Properties* (Karlheinz Schwarz, Techn. Universitat Wien, Austria, 2001).

[3] Andreev, A. V., Belov, K. P., Deriagin, A. V., Levitin, R. Z. & Menovsky, A. Magnetic and magnetoelastic properties of the UGa2 intermetallic compound. *Journal de Physique Colloques* **40 (C4)**, C4–82–C4–83 (1979).

[4] Sakon, T. *et al*. Magnetic anisotropy study of UGe2 in a static high magnetic field. *Journal of Physics: Conference Series* **51**, 255 (2006).

[5] Shorikov, A., Medvedeva, J., Poteryaev, A., Mazurenko, V. & Anisimov, V. Ab initio investigation of uranium monochalcogenides. *JETP Letters* **91**, 486–489 (2010).
